# Remarks on the Physiology of the Eye

**Published:** 1829

**Authors:** James C. Finley

**Affiliations:** Cincinnati


Art. VII.—*Remarks upon the Physiology of the Eye.*—By James C. Finley, M. D. of Cincinnati.

Having been often dissatisfied at the imperfect manner in which the physiology of vision has been treated by the most popular authors, I have thought it probable that an attempt to apply the principles of optics and physiology to some disputed points upon that subject, might not be unacceptable to the readers of the Western Journal.

It is unnecessary to speak of the structure of the eye, as this is familiar to every reader. It is sufficient to remark, that the eye is provided with two lenses which serve to concentrate the rays of light in its posterior part, so as to form an image upon the retina. Since the power of the lens depends upon its density, compared with the medium with which it is surrounded, the refractive power of the eye must principally depend upon the aqueous humour; because the chrystalline lens embodied in humours whose density is very nearly equal to its own must have its effects almost entirely counteracted by the concave surface of these humours which lie in contact with it. Hence if the chrystalline be removed an image is still formed, larger, and more indistinct, but sufficiently defined in its outlines, to serve the ordinary purposes of vision; though the range of vision is very considerably contracted.

The cornea has generally been described, as a segment of a sphere smaller than the globe of the eye. An accurate observation of the eye, however, especially in nearsighted persons, will show that the curue of the cornea deviates very considerably from that of a sphere, and as writers on optics have demonstrated, that a parabolic curve alone will so concentrate the rays of light as to form a perfect image, it is probable that nature, whose works are liable to none of the objections which arise from the imperfection of human instruments, has adopted that form which is best suited to her purposes. Scarpa, who has pointed out this fact, attributes it to the compression of the muscles of the globe of the eye. But without stopping to point out the insufficiency of this explanation, it is sufficient to remark, that nature is not often under the necessity of resorting to mechanical contrivances, to remedy the imperfection of her instruments. On the contrary, the constant pressure of the humors of the eye, acting equally in all directions, must have a tendency to remove the prominence of the cornea, and to produce that state of things which we meet with in old age.

As objects approach the eye, the point at which the rays converge is thrown further back, and consequently it has been supposed, that the eye must have the power of accommodating itself to the distance of objects, so that the image may always be formed on the retina. Before examining the truth of the various hypotheses, which have been formed in explanation of this supposed power, it would be worth while to enquire if it be really necessary.

A screen placed at the focus of a lens whose focal distance is 3-4 of an inch, will admit of an extent of vision equal to the 1-8 of an inch without impairing the distinctness of the image. The image will be larger, as the screen is removed from the focus, either to or from the lens, but its outlines will be preserved sufficiently distinct for the purpose of vision. As the object approaches the eye, the point at which the rays converge does not change more than the 1-8 of an inch, until it comes within six inches of the lens. It is difficult to make satisfactory experiments of this kind upon the eye, on account of the difficulty of removing its coats so as to obtain a distinct view of the image, without impairing its structure. If we can be allowed, however, to apply this fact in explanation of the phenomena of vision, we will discover that the eye may be adapted to a very considerable range of vision, without any power of accommodation. Without the limits of distinct vision, the eye is assisted very much by the memory in conveying a distinct impression of the object of vision, when this is familiar to us. This influence is exemplified by the effect of the imagination, in giving form and shape to those objects, which are indistinctly seen by dim twilight, or through the medium of a fog. In the same manner, when an object is suddenly placed close to the eye, the impression is confused and indistinct, and the eye accommodates itself to the distance, not suddenly, as if by muscular effort, but by gradually tracing the outlines until, by the assistance of memory and imagination, we are enabled to give them their distinct features. From the want of this assistance, we are unable to form a clear idea of the form of an unknown object that is placed out of the limits of distinct vision.

In addition to this, the eye probably has a power analogous to one possessed by the ear, of singling out the rays which proceed from a single point, and of bestowing upon them its undivided attention, and thus of forming a distinct outline, when the image upon the retina is very confused.

It is unnecessary to state to those at all acquainted with the principles of optics, that the contraction of the iris can exert no influence over the focal distance of the eye, as Richerand erroneously supposes. Its only office is to diminish the intensity of the image without affecting, as Magendie asserts, its size.

The estimate that we form of the size of objects, so far as this depends upon the eye, it is probable, depends upon what Dr. Brown has called the *muscular sense.**

---

*As many individuals are disposed to doubt the existence of a sense of this kind, it may not be improper to add, by way of illustration, a few examples of its exercise. It is by this sense we judge of the weight, shape, or size of objects, which we hold in the hand, by the resistance they offer to the muscles. By this sense we keep within the centre of gravity, and judge when we deviate so far from it as to endanger our falling. As instances of the great accuracy which it may acquire, we may witness the facility with which we direct our hand, in whatever position it may be placed, to any part of our body; and the rapidity and certainty, with which we regulate the motion of the arm, in throwing at a distant object.

There is but one point in the retina, sufficiently sensible to receive accurate impressions, and the eye in viewing an objeet, turns itself so that the rays from the different parts may be successively received upon this point. It is by the extent of motion necessary to gain a distinct view of the whole image, that we judge of its size. In viewing small objects, or those which are very familiar to us, the motions of the eye are made with such rapidity, that we do not attend to them, and appear to embrace the whole at one view; but there can be no doubt that we acquire our idea of any complex object, by a successive view of its different parts. The extent of motion necessary to embrace the whole of the object, will not be affected by the variation in the size of the image, in consequence of the rays converging to a point nearer to, or more remote from the retina, as the motions of the axis of the eye will be the same, whatever may be the imperfection of the image. If our estimate of the size of objects, depended upon the size of the image formed upon the retina, we could form no conclusion when they were brought within the point of distinct vision, as the image then very rapidly increases, fiom a two fold cause—the nearness of the object, and the imperfect convergence of the rays which proceed from it.

It is owing to the exercise of the muscles of the eye, which is necessary in viewing very near objects, on account of the imperfection of the image on the retina, that the eyes are so much fatigued in examining them.

This view of the subject, affords a very ready explanation of the fact, that we do not see objects inverted. We judge of their position, not by that of the image on the retina, but by the motion of the eye necessary to enable us to embrace the whole view. Thus we judge by the touch of the position of an object, in whatever direction the hand may be placed upon it.

The sympathy between the retina and iris is well known, and is analogous to what we observe in other parts of the system, where irritation produces contraction in distant muscles.

There is also a very close sympathy between the retina and the surface of the eye, the object of which is to produce a closure of the lids, when the eye has been too long exercised.

This, we believe, is the only instance in which inflammation is uniformly produced by irritation of a distant nerve; and what makes it more remarkable, by one that is insensible to the irritation.

This fact in combination with others of a similar nature, may permit us to question the truth of a general received opinion, that the painful sensations we experience arise from mechanical violence offered to the nerve. The pain that we experience from wounding the surface or extremities of the body is very different from that of wounding the large braches of the nerves. The heart, lungs, serous membranes and ligaments of the joints, have no sensibility to ordinary irritants, either chemical or mechanical. But they are all exquisitely sensible when inflamed; the ligaments of the joints when stretched, and the vital organs when their functions are interrupted, produce agony of the most insupportable kind. The nerves which supply these different parts produce that kind of sensation, which is necessary to give warning of interruption of function, or the approach of disease; or to guard against those injuries, to which they are most exposed; and the pain probably is produced not by mechanical violence offered to the nerve, but by the consciousness (if we may use the expression) which the nerve possesses of the suffering of the part over whose welfare it presides.

The question has been frequently proposed by physiologists; why, since the image is formed upon each of the eyes, do we not see objects double. In reply, it is generally supposed that there are corresponding points in the nerves of the two eyes, which convey a single impression to the mind. In directing our vision, instinct or experience teaches us, so to direct the eye as that the rays of light may enter in the direction of its axis. They then fall upon the most sensible part of the retina, and by moving perpendicularly to the plane of the pupil, a greater number are permitted to pass that opening, and arrive at the posterior part of the eye. Hence we acquire the habit of moving the eyes in concert, and of referring the impressions made upon these points to a single object.

If we allow this concert in action to depend upon habit, as it undoubtedly does, then there can be no other corresponding points in the eyes; because the relative place of the image will change, with every change in the distance, and position of the object.

If the rays of light therefore, enter the eye in any other direction, the object will appear double, provided, the part of the nerve in the different eyes upon which they fall, is possessed of nearly equal sensibility: Otherwise the attention is directed to the stronger impression, while the other is overlooked. Hence an object placed immediately before the eyes appears double when the vision is directed to a point nearer or more remote; or when it is brought so near that the axes of the two eyes cannot be directed towards it. When the object is placed outside of the axis of the eye, it will not appear double unless it be very brilliant, and the view be steadily directed to some other object, because the rays then fall upon a point of the retina in one eye of so little sensibility that the impression is not attended to; and it is very difficult to control the efforts of the eye to gain a distinct view.

In conformity with these principles, when an effort is made to view an object very close to the eye, two similar bodies may be so placed, that the direction of the rays of light proceeding from them, will coincide with the axes of the eyes, and consequently, an impression will be conveyed of the existence of but one object.

Something analogous to this, we have in the sense of touch. When the contiguous surfaces of the fingers are placed upon a minute object, a single idea is conveyed to the mind, but when by crossing the fingers, surfaces which are not habituated to act in concert are applied to the same object, two distinct impressions are conveyed to the mind.

It has been supposed, that the mind attends only to the impression made upon one of the eyes, and in confirmation of this opinion, an experiment of Mr. Lawrence, has been lately suggested to me.

It is alledged that when the sight is directed to an object at the distance of 15 or 20 feet, if the finger be moved before the eye, until a shade is cast over the object, it will be found that the distinct view is more obstructed when the rays of light are intercepted from one eye, than from the other; and it has been supposed from the results of this experiment, that the right eye is generally used in preference to the left.

There appears to be some mistake on this subject. The object will appear as if viewed through a semitransparent medium, when the view of either eye is intercepted, and nearly to the same extent; consequently, the indistinctness would appear to arise from the fact that in spite of our efforts to fix our attention, the finger diverts it from the other object; and this experiment may be considered as confirming the received opinion, that both eyes are used in vision.

Dec. 1829.

